# Complete Genome Sequences of Three Star-Shaped Bacteria, Stella humosa, Stella vacuolata, and Stella Species ATCC 35155

**DOI:** 10.1128/MRA.00719-19

**Published:** 2019-08-08

**Authors:** Atsushi Shibai, Tomoya Maeda, Masako Kawada, Hazuki Kotani, Natsue Sakata, Chikara Furusawa

**Affiliations:** a Center for Biosystems Dynamics Research (BDR), RIKEN, Suita, Osaka, Japan; b Universal Biology Institute, Graduate School of Science, University of Tokyo, Tokyo, Japan; Indiana University, Bloomington

## Abstract

Stella species are unique star-shaped alphaproteobacteria found in various environments. We report the complete genome sequences of three *Stella* strains, Stella humosa ATCC 43930, Stella vacuolata ATCC 43931, and *Stella* species ATCC 35155. These are the first complete genome sequences of members of the genus *Stella*.

## ANNOUNCEMENT

Although bacteria have a wide variety of cell shapes, the mechanisms underlying the generation of the complex morphology and evolution of shape diversity remain unknown. In this study, we report the first complete genome sequences of three *Stella* strains which have a unique star-shape morphology. *Stella* species are flat, six-pronged, star-shaped bacteria found in various environments, e.g., freshwater, soil, and sewage (1). The genus *Stella* comprises Gram-negative alphaproteobacteria belonging to the order *Rhodospirillales* in the family *Rhodospirillaceae* (2). These unique star-shaped bacteria were isolated first in Russia, and three species are currently deposited with the American Type Culture Collection (ATCC). Stella humosa ATCC 43930 and Stella vacuolata ATCC 43931 were isolated from a soil sample in Krasnodar and in horse manure, respectively (1). Eleven additional strains have also been isolated (3), and we deposited the third *Stella* species strain, ATCC 35155. This strain was isolated from brackish water in the Baltic Sea, and the ATCC has termed this strain Stella aquatica ATCC 35155.

*S. humosa*, *S. vacuolata*, and Stella sp. strain ATCC 35155 cells were grown in ATCC medium 1298 (brackish *Prosthecomicrobium*; https://www.atcc.org/~/media/BCF4A47455D44063A66771F94C62AF54.ashx) at 30°C and 150 rpm for 8, 3, and 6 days, respectively, and then pelleted. DNA was extracted from the cells with Wizard genomic DNA purification kits (Promega) according to the manufacturer’s instructions. Long-read library preparation for Nanopore sequencing was performed with a 1D^2^ sequencing kit (SQK-LSK308; Nanopore) without fragmentation. The libraries were then sequenced on a MinION device with a 1D^2^ flow cell (FLO-MIN107; Nanopore) and base called with Guppy v2.3.5 (Nanopore). Short-read libraries for paired-end sequencing (2 × 300 bp) were prepared using a Nextera XT kit (Illumina) without preceding fragmentation, such as ultrasonic shearing, and sequenced on an Illumina MiSeq platform with the MiSeq reagent kit v3 for 600 cycles. The number of short reads and the number and the average length of long reads for each sample are summarized in Table 1.

**TABLE 1:** General features of *Stella* species genomes and a summary of their sequencing data

ATCC no.	Bacterial species	No. of Illumina paired reads	Nanopore reads		Total coverage (×)	Genome size (bp)	GC content (%)	No. of CDSs	Accession no.
			No.	Avg length (bp)					
ATCC 43930	*Stella humosa*	2,446,912	25,819	3,340.4	140	5,832,650	69.8	5,556	AP019700
ATCC 43931	*Stella vacuolata*	3,290,870	203,511	4,874.2	330	5,834,761	70.9	5,515	AP019702
ATCC 35155	*Stella* sp.	4,069,690	186,264	3,717.6	310	5,339,096	70.6	5,058	AP019701

For *S. humosa*, the long-read assembly was performed with Canu v1.8 (4), including a read correction step. The resulting contig was then circularized with Circlator v1.5.5 (5) and polished with short reads by Pilon v1.2.3 (6). These software tools were used with default parameters. For *S. vacuolata* and Stella sp. ATCC 35155, hybrid assembly of long and short reads was performed with Unicycler v0.4.8 (7), with default parameters, including a built-in read correction step and a polishing step with Pilon. The resulting circular contigs were additionally polished with short reads by Pilon. A single contiguous circularized sequence was obtained for each of the three species. The *Stella* chromosomes were 5.3 to 5.8 Mbp and were GC rich (∼70% GC content; Table 1). Automatic annotation using the DDBJ Fast Annotation and Submission Tool (DFAST) pipeline (8) detected more than 5,000 coding sequences (CDSs) on each chromosome (Table 1).

### Data availability.

The whole-genome sequences of the three *Stella* strains were deposited with DDBJ/GenBank under accession numbers AP019700 to AP019702 (Table 1). The fastq files of raw reads were deposited in the DDBJ Sequence Read Archive (DRA)/NCBI SRA under the accession number DRA008500. The versions described in this paper are the first versions.
